# Association of *CYP2D6* polymorphisms and extrapyramidal symptoms in schizophrenia patients receiving risperidone: a retrospective study

**DOI:** 10.1186/s40780-018-0126-y

**Published:** 2018-11-19

**Authors:** Takahiro Ito, Kazuhiro Yamamoto, Fuminori Ohsawa, Ikuo Otsuka, Akitoyo Hishimoto, Ichiro Sora, Midori Hirai, Ikuko Yano

**Affiliations:** 1 Department of Pharmacy, Kobe University Hospital, 7-5-2 Kusunoki-cho, Chuo-ku, Kobe, 650-0017 Japan; 2 Department of Psychiatry, Kobe University Graduate School of Medicine, 7-5-1 Kusunoki-cho, Chuo-ku, Kobe, 650-0017 Japan

**Keywords:** Schizophrenia, Extrapyramidal symptoms, DIEPSS, *CYP2D6*, Risperidone

## Abstract

**Background:**

Risperidone is mainly metabolized by cytochrome P450 (CYP) 2D6 in the liver. The gene encoding *CYP2D6* is highly polymorphic. The average steady-state plasma concentration of risperidone active moiety is higher in the *CYP2D6* intermediate metabolizers (IMs) compared with that in the extensive metabolizers (EMs). An association between drug-induced extrapyramidal symptoms scale (DIEPSS) score and *CYP2D6* polymorphisms has not been reported to date. This study investigates the association of *CYP2D6* polymorphisms with the severity of extrapyramidal symptoms in schizophrenia patients receiving risperidone therapy.

**Methods:**

Schizophrenia patients undergoing risperidone treatment were recruited for the study in the Kobe University Hospital. We evaluated extrapyramidal symptoms of schizophrenia using the DIEPSS. CYP2D6*10 and CYP2D6*14 were analyzed using TaqMan® assays, and CYP2D6*5 was analyzed using the long-PCR method. Patients with CYP2D6*1/*5, *1/*14, *5/*10, *10/*10, and *10/*14 were classified as IMs, and patients with CYP2D6*1/*1 and *1/*10 were classified as EMs. Patients with CYP2D6*5/*5, *5/*14, and *14/*14 were classified as poor metabolizers (PMs).

**Results:**

A total of 22 patients were included in the study. No patients were classified as PMs. The dose of risperidone (mg/day) was not significantly different between EMs (*n* = 15) and IMs (*n* = 7) (median with the interquartile range: 4.0 (2.0–6.0) vs. 4.0 (2.0–7.0) mg, *p* = 0.31). The age and disease duration of schizophrenia were not significantly different between the EMs and IMs. The DIEPSS score in the IMs was significantly higher than that in the EMs (median with the interquartile range: 5.0 (3.5–6.5) vs. 0.0 (0.0–3.0), *p* < 0.001). The multiple regression analysis showed that *CYP2D6* IMs is a significant risk factor for the DIEPSS (*p* < 0.05).

**Conclusion:**

Special attentions should be paid to the onset of extrapyramidal symptoms in schizophrenia patients identified as *CYP2D6* IM undergoing risperidone therapy.

## Background

The advent of typical antipsychotic medications such as chlorpromazine revolutionized schizophrenia treatment in the 1950s [1]. Atypical antipsychotics developed after 1960s showed a relatively low frequency of extrapyramidal symptoms. The atypical antipsychotic risperidone has a high binding affinity for both dopamine D_2_ and serotonin 5-HT_2_ receptors and has a proven efficacy in the treatment of schizophrenic positive and negative symptomatology [2]. In the late years, although the atypical antipsychotics such as clozapine and risperidone that relatively had low frequency of extrapyramidal symptoms are used, many patients develop serious acute adverse effects, such as akathisia, dystonia, and parkinsonism, leading to an impaired quality of life of these patients [3–5]. In addition, insufficient management of the adverse effects due to the antipsychotics increases the patients’ mortality [6].

Risperidone is mainly metabolized to the active metabolite 9-hydroxy-risperidone by cytochrome P450 (CYP) 2D6 in the liver [7]. Previous pharmacokinetic studies of risperidone have revealed a large interindividual variability between oral dose and actual plasma concentrations [8]. Physiological factors such as age and body weight have been implicated in this variability. In addition, since the gene encoding *CYP2D6* is highly polymorphic [9], *CYP2D6* status might affect as well risperidone pharmacokinetics. Four phenotypes of *CYP2D6* have been defined as follows: (1) poor metabolizers (PMs), without enzyme activity; (2) intermediate metabolizers (IMs), with reduced enzyme activity; (3) extensive metabolizers (EMs), with normal activity; and (4) ultra-rapid metabolizers, with increased enzyme activity [9]. In the Japanese population, EMs are observed at the highest frequency and include CYP2D6*1/*1 (17.8%), *1/*2 (11.2%), *1/*10 (29.7%), and *2/*10 (7.7%) [10]. Genotypes classified into PM is rare in the Japanese population, whereas CYP2D6*1/*5 (6.3%), *5/*10 (5.2%), and *10/*10 (14.3%) classified into IM represent about one quarter [10]. CYP2D6*14 as well as CYP2D6*5 are reported as the major defective alleles found in Japanese subjects [11]. The frequencies of CYP2D6*5 and *14 were 6.2 and 2.2%, respectively [11]. The area under the concentration-time curve of risperidone in IMs is reported higher than that in EMs [12]. Findings regarding the association between *CYP2D6* polymorphisms and response to risperidone have been conflicting [13]. On the other hand, higher plasma concentrations of risperidone plus 9-hydroxy-risperidone, the active moiety of risperidone, are associated with higher incidence of adverse effects [14]. Thus, *CYP2D6* genotyping might be useful in personalizing risperidone therapy in patients with schizophrenia to reduce the incidence of adverse extrapyramidal symptoms.

A drug-induced extrapyramidal symptoms scale (DIEPSS) was developed in Japan in 1994 for evaluating the symptoms seen in psychiatry patients taking antipsychotics [15]. DIEPSS is suitable to evaluate the low incidence of extrapyramidal symptoms occurring during the treatment with atypical antipsychotics such as risperidone [16, 17]. DIEPSS was reported having high inter-rater and test-retest reliability, and a concurrent validity with other rating scales for extrapyramidal symptoms [18].

In the present study, our objective was to investigate the association of *CYP2D6* polymorphisms with the severity of extrapyramidal symptoms in schizophrenia patients receiving risperidone therapy.

## Methods

### Patients

Patients with schizophrenia defined according to the Diagnostic and Statistical Manual of Mental Disorders (DSM)-IV criteria [19] who received risperidone treatment were recruited for the study between February 2011 and July 2013 from the Kobe University Hospital. Patients were eligible for the study if DIEPSS data were available. Patient information including age, disease duration of schizophrenia, gender, body weight, laboratory data, and history of prescription was collected from the electronic medical records.

### Ethics approval

This study was designed and implemented in accordance with the Declaration of Helsinki and its amendments. The present study was approved by the Ethics Committee of the Kobe University Graduate School of Medicine for Genetic Analysis (No. 57). Written informed consent was obtained from each patient registered in the study.

### Evaluation of extrapyramidal symptoms

The extrapyramidal symptoms in each patient were evaluated using the DIEPSS by the attending psychiatrists in Kobe University Hospital when the patient received risperidone maintenance dose. Since risperidone reaches a steady-state within 2 weeks, we evaluated DIEPSS score after day 14 from the start of risperidone therapy. DIEPSS consists of one global item (overall severity) and eight individual items (gait, bradykinesia, sialorrhea, muscle rigidity, tremor, akathisia, dystonia, and dyskinesia); each item is rated on a five-point scale (0, normal; 1, minimal; 2, mild; 3, moderate; 4, severe) [15]. The primary endpoint in this study was the sum score of the nine items.

### *CYP2D6* genotyping

For each participant, we collected peripheral blood samples in EDTA tubes, which were kept at − 80 °C until use. Genomic DNA was extracted with the QIAamp DNA Blood Midi Kit® (Qiagen Inc., Valencia, CA, USA) according to the manufacturer’s instructions. CYP2D6*10 (rs1065852) and CYP2D6*14 (rs5030865) were analyzed using commercially available TaqMan® assays (Applied Biosystems, Foster, CA, USA). CYP2D6*5 (*CYP2D6* deletion) was analyzed using the long-PCR method [20]. The distinction of CYP2D6*5/*10 and *10/*10 was not analyzed in this study. On the basis of the previous study [9, 11], patients with CYP2D6*1/*5, *1/*14, *5/*10, *10/*10, and *10/*14 were classified as IMs. Patients with CYP2D6*1/*1 and *1/*10 were classified as EMs. Patients with CYP2D6*5/*5, *5/*14, and *14/*14 were classified as PMs.

### Statistical analysis

All statistical analyzes were performed using SPSS® Statistics 24.0 (IBM Japan, Tokyo, Japan). Data are expressed as the number of patients or the median with interquartile range. Fisher’s exact tests were used to test the distribution of categorical data, whereas Mann–Whitney *U* tests were employed to compare the medians of continuous values between groups. Regression analysis was performed to evaluate the relationship between risperidone dose and DIEPSS score. Multiple regression analysis was performed to identify factors affecting the DIEPSS score, in which the DIEPSS score was defined as the dependent variable, and all the independent variables (*p* < 0.2) defined in the univariate analysis were selected and tested. *P* values less than 0.05 were considered statistically significant.

## Results

A total of 33 patients were recruited from February 2011 to July 2013 and provided written informed consent. Twenty-two patients whose DIEPSS data were available were eligible for the study. Table 1 shows the demographic and clinical characteristics of 22 schizophrenia patients treated with risperidone. The CYP2D6*1/*1, *1/*5, *1/*10, and *5/*10 or *10/*10 genotypes were detected in 2, 2, 13, and 5 patients respectively. Seven patients with CYP2D6*1/*5, *5/*10, and *10/*10 were classified as IMs, and 15 patients with CYP2D6*1/*1 and *1/*10 were classified as EMs. CYP2D6*5/*5 genotype and *14 allele were not detected in any patient in this study. The dose of risperidone (mg/day) was not significantly different between EM and IM groups (median: 4.0 (2.0–6.0) vs. 4.0 (2.0–7.0) mg, *p* = 0.31). None of the patients had advanced renal dysfunction or liver dysfunction. Median levels of aspartate aminotransferase, alanine aminotransferase, and serum creatinine were in the reference ranges of our hospital for both the EM and IM groups. The age, disease duration of schizophrenia, body weight, and the number of concomitant drugs were not significantly different between EM and IM groups. Although 15 patients (68.2%) concomitantly used other antipsychotics except risperidone, the total of chlorpromazine equivalent dose (mg/day) was not significantly different between EM and IM groups (median: 500 (400–1010) vs. 475 (250–1800) mg, *p* = 0.79). In this study, olanzapine and levomepromazine were each used in six patients (27.3%), aripiprazole and chlorpromazine were each used in three patients (13.6%), quetiapine, zotepine, paliperidone, and blonanserin were each used in two patients (9.1%), and sulpiride was used in one patient (4.5%). None of the patients had a medical history of Parkinson’s disease. Paroxetine, a potent *CYP2D6* inhibitor, was concomitantly used in one patient in the EM group.

**Table 1:** Patient demographics

	EM (*n* = 15)	IM (*n* = 7)	*p*
Age (years)	33.0 (28.0–43.0)	39.0 (29.0–64.0)	0.56^a^
Disease duration of schizophrenia (years)	12.0 (6.0–16.5)	9.0 (9.0–40.0)	0.43^a^
Gender: female/male	7/8	4/3	0.50^b^
Body weight (kg)	63.0 (55.3–78.0)	64.2 (59.2–76.7)	0.96^a^
Laboratory data			
AST (IU/L)	20 (16–23)	24 (19–32)	0.31^a^
ALT (IU/L)	24 (14–33)	22 (17–94)	0.71^a^
Albumin (g/dL)	4.5 (4.3–4.7)	4.5 (4.2–4.5)	0.49^a^
Bilirubin (mg/dL)	0.60 (0.50–0.90)	0.60 (0.40–0.70)	0.32^a^
Serum creatinine (mg/dL)	0.79 (0.69–0.90)	0.74 (0.66–0.84)	0.37^a^
eGFR (mL/min/1.73 m^2^)	79.3 (70.7–91.2)	85.0 (66.7–102.6)	0.64^a^
Antipsychotics			
Risperidone dose (mg/day)	4.0 (2.0–6.0)	4.0 (2.0–7.0)	0.31^a^
Risperidone dose (mg/kg)	0.065 (0.034–0.097)	0.074 (0.053–0.112)	0.55^a^
Olanzapine (+/−)	4/11	2/5	0.65^b^
Levomepromazine (+/−)	5/10	1/6	0.35^b^
Chlorpromazine equivalent dose (mg/day)	500 (400–1010)	475 (250–1800)	0.79^a^
Other treatments			
Number of concomitant drugs	4 (2–5)	7 (2–8)	0.31^a^
Anticholinergics (+/−)	7/8	5/2	0.27^b^
Mood stabilizers (+/−)	4/11	3/4	0.39^b^
Benzodiazepines (+/−)	12/3	6/1	0.62^b^

Data are expressed as the number of patients or median with the interquartile range in parentheses

Two cases (one case in the EM group, the other case in the IM group) were excluded in the body weight and risperidone dose (mg/kg) data, because information on the body weight was not obtained

^a^Mann–Whitney *U* test

^b^Fisher’s exact test

*EM* extensive metabolizer, *IM* intermediate metabolizer, *AST* aspartate aminotransferase, *ALT* alanine aminotransferase, *eGFR* estimated glomerular filtration rate

**Fig. 1:**
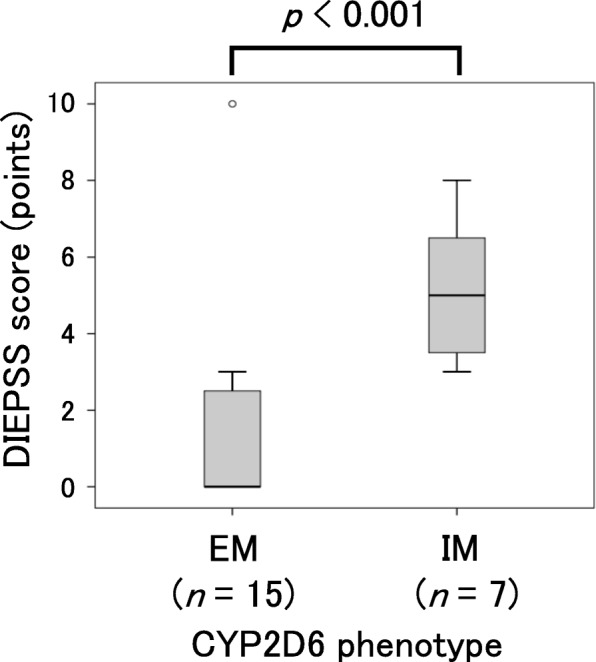
Influence of *CYP2D6* genotype on DIEPSS score. The box plot shows minimum, first quartile, median, third quartile, and maximum. The central rectangle spans the first quartile to the third quartile (the interquartile range). An outlier between 1.5 and 3 times the interquartile range is shown as an open circle. Statistical analysis was performed using the Mann–Whitney *U* test

**Fig. 2:**
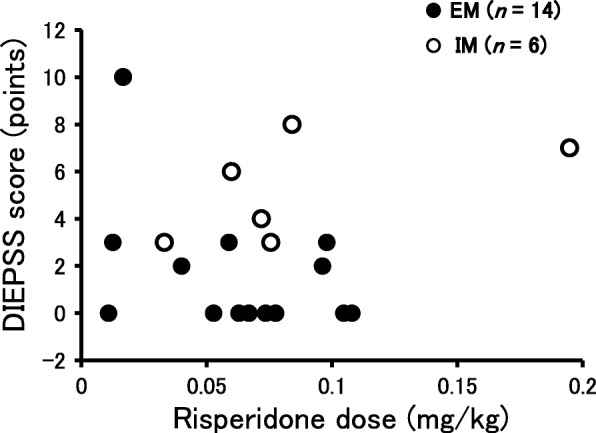
Correlation between risperidone dose per body weight and DIEPSS score. Two cases were excluded because information on the body weight was not obtained

Onset of extrapyramidal symptoms (DIEPSS score ≥ 1) was observed in 13 (59.1%) out of the 22 patients. The DIEPSS score in the IM group was significantly higher than that in EM group (Fig. 1; median: 5.0 (3.5–6.5) vs. 0.0 (0.0–3.0), *p* < 0.001). No significant correlation was observed between the DIEPSS score and risperidone dose corrected by body weight when all patients were included in the analysis. (Fig. 2; y = 5.55x + 2.31, R^2^ = 0.006, *p* = 0.749). Also, no significant correlation was observed between the DIEPSS score and risperidone doses (mg/kg) when the regression analysis is conducted for each genotype (Fig. 2; y = − 34.33x + 3.80, R^2^ = 0.176, *p* = 0.136 in the EM group, y = 20.85x + 3.36, R^2^ = 0.297, *p* = 0.263 in the IM group).

Multiple regression analysis was performed to identify factors affecting the DIEPSS [DIEPSS = 3.81 + 0.043 × age (years) − 4.15 × bilirubin (mg/dL) − 4.77 × serum creatinine (mg/dL) + 2.65 × *CYP2D6* status (IM = 1 and EM = 0)] (Table 2). *CYP2D6* IM was proven to be a significant factor affecting the DIEPSS (*p* < 0.05).

**Table 2:** Predictive factors for the DIEPSS score

Factors	B	Std. error	*p*
Constant	3.81	4.10	0.367
Age	0.043	0.037	0.265
Bilirubin	−4.15	2.82	0.160
Serum creatinine	−4.77	4.23	0.276
*CYP2D6* IM	2.65	1.16	0.035^*^

The *F* score of this model was 4.24 (*p* = 0.016) with adjusted *R*^2^ = 0.393

*B* unstandardized coefficients, *IM* intermediate metabolizer

^*^ *p* < 0.05 was considered statistically significant

## Discussion

Among 22 patients, extrapyramidal symptoms (DIEPSS score ≥ 1) were determined in 13 patients (59.1%), demonstrating a strong association between adverse drug effects and the risperidone therapy, opposite to the relatively low frequency reported.

The DIEPSS score in the *CYP2D6* IM group was significantly higher than that in EM group in this study, while we found no difference in risperidone dosing corrected by body weight between the two groups. Since adverse reactions can be correlated with risperidone concentrations, the 2017 consensus guidelines in neuropsychopharmacology recommend therapeutic drug monitoring for patients treated with risperidone [21]. To avoid neurological adverse reactions, risperidone doses which achieve over 40 ng/mL risperidone plus 9-hydroxy-risperidone plasma concentration should only be administrated in cases of insufficient or absence of therapeutic response [21]. Unfortunately, we did not perform plasma concentration measurements of risperidone and the subsequent dose adjustments in this study. On the basis of simulation results in a typical patient (body weight of 70 kg and creatinine clearance of 120 mL/min) on stable therapy with risperidone 2 mg twice daily (4 mg/day), the average steady-state plasma concentration of risperidone active moiety in the IM group is approximately 40 ng/mL and 1.6-fold higher compared with patients with CYP2D6*1/*1 genotype [8]. Although pharmacokinetics of risperidone and its active metabolite are affected by age, gender, body weight, smoking habits, co-administered drugs, and *CYP2D6* genotype [14], the patient characteristics such as age, body weight, and laboratory data were not significantly different between EM and IM groups in this study. Disease duration of schizophrenia can affect the severity of extrapyramidal symptoms, but there were no significant differences between EM and IM groups. Additionally, the results in this study were similar even if one patient who used paroxetine was excluded (data not shown). Therefore, we speculate that the decrease in the clearance of risperidone in the IM group causes an increased risperidone plasma concentration, which increases the severity of the extrapyramidal symptoms. This is the first report to show an association between DIEPSS score and *CYP2D6* polymorphism.

In this study, no significant correlation was obtained between the DIEPSS score and risperidone dose corrected by the body weight when the regression analysis is conducted for all patients or each genotype. A previous study showed a weak but significant correlation between oral risperidone dose and plasma concentrations of the active moiety [22]. On the other hand, pharmacokinetics of risperidone and its active metabolite are affected by age, gender, body weight, smoking habits, and co-administered drugs in addition to *CYP2D6* genotype [14]. Therefore, we consider that the sum of plasma concentrations of risperidone and 9-hydroxy-risperidone cannot be precisely predicted by the risperidone dose only. Moreover, we consider that the sample size in this study might be insufficient to detect the relationship between the DIEPSS score and risperidone doses, and there might be other risk factors of higher DIEPSS to explain why the DIEPSS score and risperidone dose are not correlated in our study.

The results of multiple regression analysis show that *CYP2D6* polymorphism is the only significant factor to be correlated with the DIEPSS. Since the sample size in this study is relatively small, any effects of other previously reported factors influencing the pharmacokinetics and pharmacodynamics of risperidone remain to be clarified with a larger number of patients.

Several limitations of this study should be acknowledged. This study is a retrospective study based on medical records, and we did not measure the plasma concentration of risperidone and active metabolite 9-hydroxy-risperidone. In a future prospective study, it is necessary to clarify the relationship among *CYP2D6* polymorphism, plasma concentrations of risperidone and its metabolite, pharmacological effects, and the severity of adverse effects in Japanese patients. In addition, detailed information on the duration from the start of risperidone to scoring DIEPSS was not available in this study, and 15 patients (68.2%) concomitantly used antipsychotics beside risperidone. Therefore, a prospective study should be conducted including patients on newly risperidone monotherapy to exclude the potential influence of the duration of risperidone treatment and other antipsychotics on the result interpretation.

## Conclusions

The DIEPSS score was significantly higher in the *CYP2D6* IM group compared with in the EM group. The role of *CYP2D6* genotyping in personalizing risperidone therapy in patients with schizophrenia should be examined in a prospective study using plasma concentration measurements of risperidone and 9-hydroxy-risperidone.

## Abbreviations

ALT: Alanine aminotransferase
AST: Aspartate aminotransferase
CYP: Cytochrome P450
DIEPSS: Drug-induced extrapyramidal symptoms scale
DSM: Diagnostic and Statistical Manual of Mental Disorders
eGFR: Estimated glomerular filtration rate
EMs: Extensive metabolizers
IMs: Intermediate metabolizers
PMs: Poor metabolizers
